# Maximizing Antioxidant Potential in Picual Virgin Olive Oil: Tailoring Agronomic and Technological Factors with Response Surface Methodology

**DOI:** 10.3390/foods13132093

**Published:** 2024-07-01

**Authors:** Antonia de Torres, Francisco Espínola, Manuel Moya, Cristóbal Cara Corpas, Alfonso M. Vidal, Salvador Pérez-Huertas

**Affiliations:** 1 Department of Chemical, Environmental and Materials Engineering, Higher Polytechnic School of Linares, University of Jaén, University Avenue (Southern Belt), 23700 Linares, Spain; antorres@ujaen.es (A.d.T.); ccara@ujaen.es (C.C.C.); 2 Department of Chemical, Environmental and Materials Engineering, University of Jaén, Paraje Las Lagunillas, B-3, 23071 Jaén, Spain; fespino@ujaen.es (F.E.); mmoya@ujaen.es (M.M.); amvidal@ujaen.es (A.M.V.); 3 Department of Chemical Engineering, Faculty of Science, University of Granada, Av. Fuente Nueva S/N, 18001 Granada, Spain

**Keywords:** malaxation, response surface methodology, virgin olive oil, polyphenols, tocopherols, cultivar

## Abstract

Over the past years, a prolonged drought has affected Spain, raising significant concerns across various sectors, especially agriculture. This extended period of dry weather is profoundly affecting the growth and development of olive trees, potentially impacting the quality and quantity of olive oil produced. This study aims to assess the impact of agronomic factors, i.e., olive maturation and irrigation management, as well as the technological factors involved in the production process, on the antioxidant content of Picual virgin olive oil. Mathematical models were developed to maximize the concentration of polyphenols, orthodiphenols, chlorophylls, carotenes, and tocopherols in olive oils. Findings indicate that increasing the malaxation temperature from 20 to 60 °C and reducing the mixing time from 60 to 20 min positively influenced the polyphenol and orthodiphenol content. Although irrigation did not significantly affect the polyphenols, pigments, and α-tocopherol contents, it may enhance the β- and γ-tocopherol content. Optimal conditions for producing antioxidant-enriched virgin olive oils involved olives from rainfed crops, with a moisture index of 3–4, and a 60-min malaxation process at 60 °C. Under these conditions, the total phenol content doubled, pigment content increased fourfold, and α-tocopherol content rose by 15%. These findings provide relevant knowledge to interpret the year-to-year variation in both organoleptic and analytical profiles of virgin olive oils.

## 1. Introduction

The Mediterranean diet is widely recognized as one of the leading dietary regimens. It is associated with preventing degenerative diseases and prolonged longevity. The most characteristic component of the Mediterranean diet is olive oil, especially virgin olive oil. Virgin olive oil is composed of two primary fractions, i.e., the saponifiable fraction, formed mainly by major constituents such as triglycerides and phosphoglycerides, and the unsaponifiable fraction, which consists of minor constituents. The latter fraction constitutes up to 2% of the total composition and is a complex mixture of approximately 230 compounds [1]. Some of these compounds, such as polyphenols, photosynthetic pigments, and tocopherols, are responsible for the innumerable health benefits associated with virgin olive oil [2]. For instance, the phenolic content plays a crucial role in the health-promoting qualities of virgin olive oil [3]. In recent years, numerous in vitro and animal studies have shown that virgin olive oil phenolic extract possesses anticarcinogenic properties [1,4,5]. Epidemiological studies have also shown that consuming polyphenol-rich virgin oil provides a cardioprotective effect [6]. However, this beneficial effect is related to the concentration of these compounds in virgin olive oil, which is strongly influenced by technological and agronomic factors, such as the cultivar, degree of maturation, and irrigation [7,8,9].

Photosynthetic pigments found in virgin olive oil can be categorized into two groups: chlorophylls and pheophytins, and carotenoids. Chlorophylls are the pigments responsible for the green coloration of virgin olive oils. Their molecular structure comprises four pyrrolic rings, each coordinated by an Mg^2+^ ion, forming a highly stable planar complex. In acidic conditions, the Mg^2+^ ion is replaced by hydrogen, leading to the formation of pheophytins. The content of chlorophylls and pheophytins in virgin olive oils may vary between 20 and 120 mg/kg, depending on agronomic factors. Notably, chlorophylls and pheophytins exhibit a pro-oxidant effect on lipids when exposed to light, while in the absence of light, they act as antioxidants [10]. Carotenoids are responsible for the yellow coloring of virgin olive oils. They are unique natural tetraterpenes formed from the union of eight isoprene units, creating a 40-carbon atom skeleton. The total carotenoid content in Spanish virgin olive oils typically ranges from 3 to 20 mg/kg, with lutein and β-carotene being the predominant types. Consuming foods rich in carotenoids has been associated with a reduced cancer risk [11]. Studies have suggested that lutein can help prevent age-related macular degeneration and cataract formation [12]. Additionally, carotene is a vital provitamin A source [13].

Tocopherols are other relevant bioactive components present in olive oil. These compounds are considered the most important natural antioxidants soluble in lipids [14]. Virgin olive oil contains α-, β-, and γ-tocopherols, with the former being the most abundant, accounting for over 95% of the total tocopherol content [15]. Alpha-tocopherol is widely recognized as the most active form of vitamin E in mammals. It acts as a potent antioxidant, capable of inhibiting lipid oxidation in both food and the human body [16]. Thus, α-tocopherol not only contributes to the nutritional value of virgin olive oil but also enhances its stability, providing protection against oxidation. The concentration of tocopherols in virgin olive oils typically varies from 50 to 400 mg/kg. As the previous compounds, it is highly influenced by agronomic factors, including cultivar, fruit ripeness index, and agroclimatic conditions. For example, Uceda et al. [17] found that the cultivar is the most important factor affecting the total tocopherol content. In addition, Beltrán et al. [18] stated that the ripeness index significantly influences the tocopherol content of olive oil. According to their findings, an increase in the fruit ripening leads to a decrease in α- and β-tocopherol levels, while γ-tocopherol levels increase. Therefore, it is essential to tailor the agronomic factors to increase the amount of functional compounds in olive oil, thereby enhancing its health-promoting properties.

It is worth mentioning that a prolonged drought has affected Spain during the last few years. The adverse effects of this drought on the growth and development of olive trees have prompted research into its impact on the quality and quantity of olive oil produced. According to the Federación de Asociaciones de Consumidores y Usuarios de Andalucía (FACUA), supermarket oil prices in Spain have risen by almost 110% since 2022 [19]. Hence, monitoring the climatic change effects on olive oil features becomes imperative to provide valuable insights into mitigating drought consequences and ensuring the resilience of this vital agricultural sector in Spain. In the meanwhile, numerous researchers have identified the milling and malaxation stages as the most critical in determining the oil quality [20]. According to de Torres et al. [21], monitoring the temperature, duration, and amount of talc added to the olive paste during the malaxation is essential as a technological aid to obtain an oil with exceptional organoleptic qualities. In light of these facts, this study aimed to explore the influence of the technological factors involved in olive paste processing, including temperature, time, and dosage of coadjuvant, on the concentration of total polyphenols, orthodiphenols, photosynthetic pigments, and tocopherols in the olive oil. Additionally, the impact of olive maturity and cultivation methods on the levels of these natural antioxidants was also evaluated. For this purpose, Picual variety olives at different ripening stages and cultivated under different growth conditions, i.e., rainfed and irrigated, were used. Finally, mathematical models were developed to determine the optimal operational conditions for maximizing the antioxidant content in virgin olive oils.

## 2. Materials and Methods

### 2.1. Samples

Picual olive fruits (*Olea europaea* L.) cultivated in the province of Jaén (Spain) and harvested from October to the end of December (2021) were used. In total, eight sets of olive samples (10 kg each) were collected at different stages of maturity and from both rainfed and irrigated crops, i.e., rainfed olives with a maturity index (MI) of 1.6, 3.1, 4.0, and 5.3 and irrigated olives with an MI of 1.2, 2.8, 3.8, and 5.5. The maturity index (MI) was determined following the procedure described elsewhere [22]. All olive samples were characterized by measuring their moisture content, solid content, and oil percentage using the Soxhlet method.

### 2.2. Oil Extraction

Oil extraction was performed on the same day as harvesting. The malaxation process was carried out by varying the temperature (20–60 °C), time (20–60 min), and talc dosage (0.5–2 wt.%) for each of the eight olive samples under study. Oil samples were obtained using the Abencor system (Abencor analyser, MC2 Ingeniería y Sistemas S.L., Seville, Spain) under laboratory conditions. This equipment is commonly used to simulate the industrial process of producing olive oil. This method encompasses grinding, mixing, and centrifugation. The extracted oils were allowed to settle in a graduated tube for approximately 3–4 h, subjected to paper filtration, and stored under a nitrogen atmosphere at −18 °C until they were analyzed.

### 2.3. Chemicals

All solvents used in this study were of HPLC quality (Sigma-Aldrich, St. Louis, MO, USA). The water utilized was of Milli-Q quality (Millipore Corp, Bedford, MA, USA). Standards for α-, β-, and γ-tocopherol were purchased from Calbiochem (San Diego, CA, USA).

### 2.4. Virgin Olive Oil Analysis

#### 2.4.1. Determination of Total Polyphenols

The total polyphenol content in olive oil samples was determined using the method described by Vázquez et al. [23]. Briefly, a sample of 10.00 ± 0.01 g of filtered oil was dissolved in 50 mL of hexane. The polyphenolic fraction was isolated by three liquid–liquid extractions using 20 mL of a methanol–water mixture (60:40 *v*/*v*). The extracts were collected in a 100 mL volumetric flask and diluted to the mark with distilled water. To a 50 mL volumetric flask, 5 mL of the methanolic extract, 35 mL of distilled water, and 2.5 mL of Folin-Ciocalteau reagent were added. The mixture was stirred, and 5 mL of a saturated sodium carbonate solution was added. Finally, the mixture was made up to the mark with distilled water. This solution was left to stand for 60 min in the dark, and the absorbance was measured at 725 nm using cells with a 1 cm light path. The same solution was employed as a blank but without the addition of the polyphenol extract. Absorbance was measured using a Biochrom spectrophotometer. Quantification was carried out using a standard curve with caffeic acid, and the results are expressed in mg of caffeic acid per kg of oil.

#### 2.4.2. Determination of Orthodiphenols

The orthodiphenols in the olive oil samples were determined using the method described by Gómez-Alonso et al. [24]. Five mL of the methanolic extract was mixed with 1 mL of a 5% sodium molybdate dihydrate in ethanol/water (1:1 *v*/*v*). Simultaneously, a blank was prepared by mixing 5 mL of the extract and 1 mL of 50% ethanol in distilled water. The sample and blank were agitated for 2 min using a vortex mixer and left to stand for 15 min. After this, the absorbance was measured at 370 nm using a Biochrom spectrophotometer. Quantification was performed by employing a calibration line from caffeic acid, and the results were expressed in mg of caffeic acid per kg of oil.

#### 2.4.3. Determination of Photosynthetic Pigments (Chlorophylls and Carotenoids)

To determine the total photosynthetic pigments, the method proposed by Mínguez et al. [25] was followed. Absorbance was measured using cyclohexane as a solvent at wavelengths of 670 nm and 470 nm for chlorophyll and carotenoid pigments, respectively.

#### 2.4.4. Determination of Tocopherols

IUPAC method No. 2432 was utilized to determine the tocopherol content. In short, 1.50 ± 0.01 g of oil was diluted to a volume of 10 mL, using the same mobile phase as the chromatographic analysis, which consisted of 0.5% isopropanol in n-hexane. A high-pressure liquid chromatography (HPLC) system (Shimadzu Corp., Kyoto, Japan) equipped with a binary pump (model 1525) was used. It featured an automatic self-cooled injector (model 2707) and a detector with a photodiode array (model 2998), providing a signal at a wavelength of 296 nm. A Lichrospher Si 60 column with a particle size of 5 μm was used. Chromatographic separation was conducted in isocratic mode with a mobile phase consisting of 0.5% isopropanol in n-hexane. The elution flow rate was 1 mL/min, the oven temperature was maintained at 35 °C, and the injection volume was 20 μL. Tocopherols were quantified using calibration lines of standard tocopherol solutions (α, β, and γ), and the results were expressed in mg/kg of oil.

### 2.5. Experimental Design and Statistical Analysis

Statistical design of experiments (SDE) can be defined as a methodology based on statistical tools for planning and analysis [26]. It involves planning the optimal experimental strategy to obtain the desired information with minimum cost and then analyzing the experimental results to ensure the highest reliability in the conclusions drawn [27]. A central composite design (CCD) with four central points was employed to conduct this study. This design allows examination of the influence of the olive paste processing conditions (factors), including temperature, time, and talc dosage, on the total content of polyphenols, orthodiphenols, chlorophylls, carotenes, and tocopherols (α, β, and γ) in the oil samples. In this design, five levels were studied for each factor, i.e., −α, −1, 0, +1, +α, where the coded value (−1) corresponds to the lower limit and (+1) to the upper limit of the actual value. Axial points (α) were calculated based on the number of factors, with a value of 1.68 for three factors. Factors are typically coded to provide a uniform framework for investigating their effects and to allow for easy comparison of coefficients in the models. By applying the central composite rotatable design (CCRD), a total of 18 experiments were required, varying the temperature (20–60 °C), time (20–60 min), and talc dosage (0.5–2%) for each of the eight olive samples studied. Therefore, a total of 144 samples were analyzed. Table 1 displays the experimental design and the actual and coded values of the factors used in each experiment.

The experimental data for each response were processed using Design-Expert software, version 8.7.1 (Stat-Ease, Inc., Minneapolis, MN, USA), and analysis of variance (ANOVA) was used to determine the model determination coefficients. A quadratic model was designed for each response as per Equation (1) to describe the behavior of the response data:

Y = β_0_ + β_1_ T + β_2_ t + β_3_ C + β_12_ T t + β_13_ TC + β_23_ t C + β_11_ T^2^ + β_22_ t^2^ + β_33_ C^2^ ± SD (1)

where T is the temperature (°C), t is the time (min), and C is the talc dosage (%). The predicted response (Y) is correlated with the set of coefficients (β): the intercept (β_0_), linear (β_1_, β_2_, β_3_), interaction (β_12_, β_13_, β_23_), and quadratic (β_11_, β_22_, β_33_). Finally, SD is the standard deviation.

Statistical significance of the model and model coefficients were determined at the 5% probability level (*p*-value ≤ 0.05). The response models were expressed in terms of coded factors, excluding statistically insignificant terms. The factors were coded using the following transformation:

(2) $$x_{i}=\frac{X_{i}-X_{0}}{\Delta X}$$

where x_i_ represents the dimensionless coded value of factor X_i_, X_0_ is the value of X_i_ at the center point or average level of the factor, and ΔX represents half of the step change.

## 3. Results and Discussion

### 3.1. Olive Characterization

Table 2 presents the results of MI, moisture, oil, and solid contents of the olive samples. Generally, olives from rainfed crops produced higher oil percentages than those from irrigated crops, possibly due to the larger moisture content in the latter. The olive oil content increased with the increase in MI. In contrast, there is no clear tendency for moisture and solids contents. This can be attributed to the prolonged drought in Spain and the irregular rainfall during the harvest period.

The experimental results of total polyphenols, orthodiphenols, chlorophylls, carotenes, and tocopherols (α, β, and γ) content in oil samples obtained from olives cultivated under irrigated and rainfed conditions are shown in Table 3 and Table 4. For a meaningful comparison, the selected samples had a similar maturity index, i.e., 3.8 and 4. It should be noted that the tables and figures presented in this paper are merely representative examples of all the results obtained from eight different olive oils, each subjected to 18 runs, resulting in a total of 144 samples.

It can be clearly seen that the α-tocopherol content in the analyzed samples was significantly higher than that of β-tocopherol and γ-tocopherol, regardless of the cultivation method. In both cases, the α-tocopherol content was approximately 360 mg/kg, whereas the content of β- and γ-tocopherol was about 5 and 20 mg/kg, respectively. This is consistent with previous research indicating that Picual virgin olive oil contains significant levels of α-tocopherol, with minimal traces of β- and γ-tocopherols [28,29]. Similar findings were obtained for the samples derived from olives with a different maturity index. Those results were used in Equation (1) to calculate the mathematical coefficients of the prediction models, which are discussed as follows.

### 3.2. Malaxation Conditions

**Figure 1:**
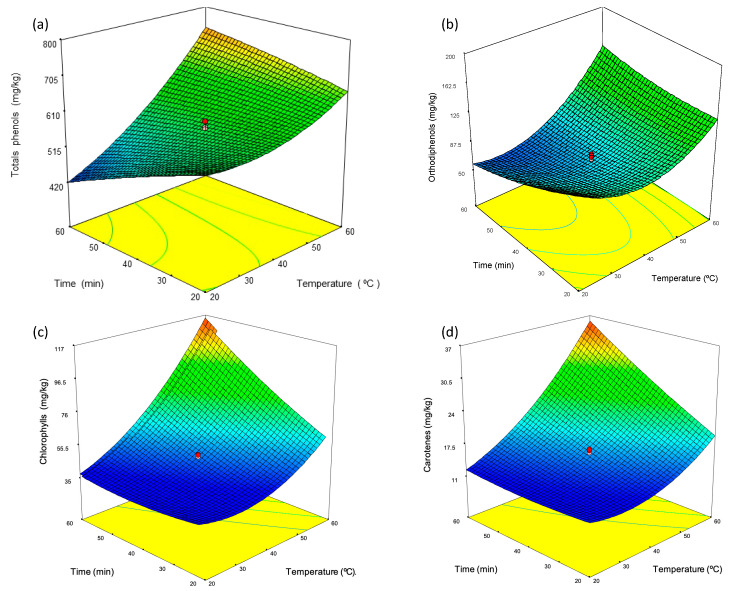
Response surfaces of (**a**) phenols (irrigation—M.I 3.8), (**b**) orthodiphenols (rainfed—MI 3.1), (**c**) chlorophylls (rainfed—M.I 1.6), and (**d**) carotenes (rainfed—M.I 1.6).

**Figure 2:**
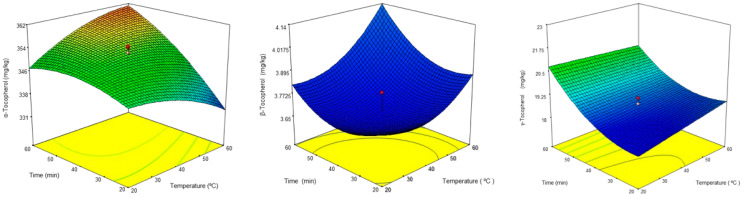
Response surfaces of α-tocopherol (irrigation—M.I 3.8), β-tocopherol (irrigation—M.I 3.8), and γ-tocopherol (irrigation—M.I 3.8).

An example of one of the models developed for the optimization of the malaxation process and the enrichment of the target compounds is given in Table S1. These models were obtained by processing the experimental data using Design-Expert software. The ANOVA analysis shows that all models are statistically significant (*p*-value ˂ 0.05). Additionally, models were validated based on a high coefficient of correlation, with an R^2^ value greater than 0.95 in most cases (Tables S1 and S2). In general, the models indicate that the malaxation temperature was the most significant factor for polyphenols and orthodiphenols, positively affecting the content of these compounds in the oil samples (Table S1). Conversely, malaxation time negatively influenced the content of these compounds, although this effect was generally less significant than that of temperature. The models also indicate a minimal influence of the talc dose on the levels of these compounds. Thus, it can be assumed that the impact of talc dose on the concentration of polyphenols and orthodiphenols in olive oil was not relevant. The response surfaces were used to determine the optimal output values (compound content) and related input values (temperature, time, and talc dosage). The surface plots with the optimal conditions for achieving the highest yield of the target compounds are presented in Figure 1 and Figure 2.

Figure 1a displays the response surface of the influence of malaxation temperature and time on the total phenol content in oil samples extracted from olives with an MI of 3.8 and grown under irrigation conditions. The surface shows a positive interaction between temperature and time, indicating that the influence of time becomes more positive at higher temperatures. The optimal malaxation conditions to produce oils with a high phenolic content involved a duration of 60 min and a temperature of 60 °C. Under these conditions, the total phenol content increased twofold from 420 to almost 800 mg/kg. For orthodiphenols, the temperature was found to be the most relevant variable in the malaxation process (Figure 1b). Higher concentrations of orthodiphenols (145–165 mg/kg) were achieved by conducting malaxations at temperatures exceeding 40 °C, regardless of time. Similar results were obtained for oils extracted from olives cultivated under different conditions (see Figure S1). It is worth noting that the total phenol concentration found in this study was significantly higher than those reported in previous studies. For instance, Parenti et al. [30] studied the effect of malaxation temperature on the total phenol content in virgin olive oil from the Frantoio variety. The highest phenol concentration of 180 mg/kg was reported at a malaxation temperature of 27 °C. Trombetta et al. [31] studied the phenol content in Italian virgin olive oils obtained from Frantoio and Casaliva cultivars, reporting the highest phenol content of 209 and 326 mg/kg, respectively. In another study, Veneziani et al. [32] found concentrations of 213, 687, and 393 mg/kg in oils obtained from three Italian cultivars, i.e., Canino, Moraiolo, and Peranzana, using low malaxation temperatures (below 25 °C). The differences in the phenolic content between the oils analyzed in this study and those from previous research can be attributed to the tailored malaxation conditions employed, along with the different olive growing areas, crop year, and fruit varieties.

In the case of photosynthetic pigments, the temperature was again the most significant factor, while the dose of talc had almost no influence. As an example, Figure 1c,d displays the response surfaces for the chlorophylls and carotenes models. These surfaces correspond to oil samples obtained from olives with an MI of 1.6 and cultivated under rainfed conditions. As can be seen, both pigments exhibited a similar response surface, indicating that the impact of the malaxation conditions on chlorophyll and carotene contents was quite similar. Higher pigment contents were achieved by conducting long malaxation processes (50–60 min) at high temperatures (50–60 °C). Under conditions yielding the highest pigment levels, the concentration of chlorophylls and carotenes increased approximately fourfold, from 30 to 120 mg/kg and from 10 to 37 mg/kg, respectively. Malaxation temperatures below 50 °C were not effective, regardless of the malaxation time or talc dose. Similar results were obtained for oils extracted from olives at different ripening stages (Figure S1). These findings are consistent with the previous studies by Espínola et al. [33] and Vidal et al. [34], which were carried out using Picual virgin olive oil extracted from olives harvested between 2005 and 2016.

Regarding the tocopherols, the models suggest that all factors positively influenced the response variable (Tables S1 and S2). However, this influence was relatively small compared to the magnitude of the independent term. Consequently, the variation in the olive oil tocopherol content underwent minor modifications throughout the experiments. This is visible in Figure 2 (left), which shows the α-tocopherol response surface for oils extracted from olives with an MI of 4 and cultivated under rainfed conditions.

Figure 2 (left) shows that the concentration of α-tocopherol ranged between 325 and 362 mg/kg. Moreover, it illustrates that at higher temperatures, the α-tocopherol concentration decreased as the malaxation time decreased. The highest concentration of α-tocopherol was obtained by conducting a malaxation process for 60 min at 60 °C. Additionally, as in previous cases, the talc dose did not significantly influence the α-tocopherol content (see Figure S1d). The α-tocopherol content obtained in the oils of this study is comparable to that reported by other authors. For instance, Inarejos-García et al. [20] studied the influence of malaxation conditions on the quality of Cornicabra virgin olive oils and found that the oils subjected to malaxation at 40 °C for 60 min exhibited the highest α-tocopherol concentration of 225 mg/kg. Peres et al. [35] examined the α-tocopherol content in virgin oils obtained from Portuguese olive cultivars, specifically Cobrançosa and Galega Vulgar, at different ripening stages. The highest α-tocopherol concentrations found in Cobrançosa and Galega Vulgar samples were 224 and 243 mg/kg, respectively. Trombetta et al. [31] studied the α-tocopherol content in 10 Italian virgin olive oils obtained from Frantoio and Casaliva cultivars and reported α-tocopherol contents of 252 and 280 mg/kg, respectively.

The oil samples used for the α-tocopherol study were also analyzed for β- and γ-tocopherol content. The models for these compounds show low coefficients for most terms, indicating that the impact of the factors on the response was not very significant (Table S1). This low influence is visible in Figure 2, where the concentration of β-tocopherol remained almost constant despite variations in temperature and time. In the case of γ-tocopherol, the highest concentration was achieved by malaxation for 60 min at the lowest temperature, i.e., 20 °C (Figure 2). Contrary to the previous cases, increasing the temperature had a negative effect on the γ-tocopherol content. Nevertheless, Picual virgin olive oil has a significantly lower β- and γ- tocopherol content compared to the other compounds studied (Table 3 and Table 4). Therefore, it can be concluded that the optimal malaxation conditions to produce antioxidant-enriched oils involved a duration of 60 min and a temperature of 60 °C, regardless of the talc dosage. Under these conditions, the total phenol content doubled, pigment content increased approximately fourfold, and the α-tocopherol content increased by 10–15%, while the β- tocopherol showed a slight increase.

### 3.3. Fruit Maturation

In this section, the influence of fruit ripeness on the oil antioxidant content was studied. For this purpose, the maximum value of each response determined by the models was used. Table 5 shows the variation of total phenol and orthodiphenol concentration as a function of the maturity index and irrigation management. The malaxation conditions used to reach the maximum values are also shown in the table.

For irrigated crop samples, the phenol concentration increased with increasing the MI up to 2.8 and decreased thenceforth. The highest phenol concentration, i.e., 760.9 mg/kg, was achieved for the oils obtained from olives with an MI of 2.8 and subjected to a malaxation process of 60 min, 60 °C, and 0.96% talc dose. A ripeness index larger than 3.8 was found to be inefficient, as it led to a significant decrease in the total phenol content (Table 5). The decrease in phenolic concentration observed as the fruit matured may result from the higher activity of endogenous enzymes occurring during the ripeness process. Peres et al. [35] also reported a significant decrease in total polyphenol content for a maturity index greater than 3.3 in olive oils obtained from Cobrançosa and Galega Vulga varieties. Furthermore, Tang et al. [36] reported similar findings using olives from Picual, Empeltre, Arbequina, and Manzanilla varieties harvested in China with a maturity index ranging from 2 to 4. Concerning rainfed crop samples, the MI did not significantly influence the total phenol content (±6% mg/kg). The oil extracted from olives with an MI of 3.1, cultivated under rainfed conditions, and subjected to a maxalation temperature of 60 °C for 60 min with 2% talc dosage yielded the highest phenol concentration, i.e., 771 mg/kg. The variation in orthodiphenol content with the maturity index followed a similar pattern to that of total polyphenols. The highest concentration of orthodiphenol, 175 mg/kg, was obtained from oils extracted from olives grown under the same conditions as those producing the highest concentration of phenols (Table 5). This would allow for their simultaneous enrichment. The concentrations of chlorophylls and carotenes as a function of the maturity index are given in Table 6.

The pigment contents decreased with increasing maturity index, regardless of cultivation condition. Thus, the pigment degradation was not significantly affected by the irrigation conditions. For both compounds, the highest concentration was found for oils derived from olives with an MI of 1.6, cultivated under rainfed conditions and subjected to a malaxation process for 60 min at 60 °C. Under these conditions, the concentrations of chlorophylls and carotenes were 117 and 36 mg/kg, respectively. Increasing the MI from 1.6 to 5.3 reduced the chlorophylls and carotenes concentration by 40% and 50%, respectively. Similar findings were found by Criado et al. [37] in oils derived from Arbequina and Farga varieties, who reported a loss of pigmentation during fruit ripening.

Table 7 shows the variation of tocopherol concentration as a function of the maturity index and irrigation management. As for the malaxation study, the quantitative variation of all tocopherols was less significant compared to the other compounds. For instance, the concentration of α-tocopherol ranged from 369 to 382 mg/kg for samples obtained from rainfed olives with MI values of 1.6 and 5.3, respectively. For oils derived from irrigated olives, the concentration of α-tocopherol increased from 336 to 352 mg/kg for samples with an MI of 1.2 and 2.8, respectively, and decreased to 346 mg/kg for samples with an MI of 5.5.

Concerning the β-tocopherol content, it decreased from 8.5 to 4.9 mg/kg as the olive maturated for the oils derived from irrigated crops. However, the oils obtained from rainfed crops remained almost constant. The γ-tocopherol content increased with olive fruit maturity, regardless of the cultivation conditions. The highest concentration of γ-tocopherol (24.1 mg/kg) was found for the samples with the highest MI. The concentrations of β- and γ-tocopherol obtained in this study were twofold larger than those reported by Beltrán et al. [18] and Peres et al. [35] for the Hojiblanca and Galega Vulgar and Cobrançosa varieties. These results show that by tailoring the appropriate ripening stage of the fruit, it is possible to produce olive oils with increased polyphenol and tocopherol levels, thereby enhancing their functional properties to a certain extent.

### 3.4. Irrigation Management

**Figure 3:**
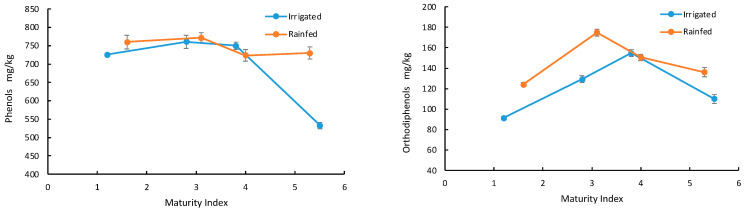
Polyphenol (**left**) and orthodiphenol (**right**) content in oils extracted from olives at different MI and grown under rainfed and irrigated conditions.

Figure 3 shows the impact of irrigation on the total phenol and orthodiphenol content in oils at different fruit ripening stages.

**Figure 4:**
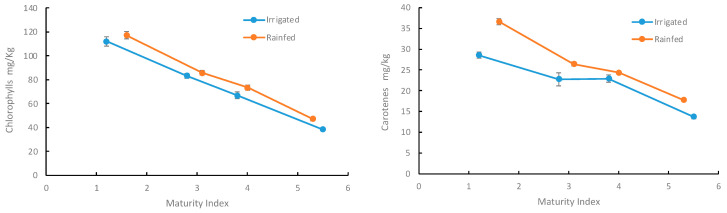
Chlorophyll (**left**) and carotene (**right**) content in oils extracted from olives at different MI and grown under rainfed and irrigated irrigation.

In general, the content of polyphenols was slightly higher in oils extracted from olives cultivated in dry conditions. Furthermore, oils obtained from rainfed crops showed a 20–25% increase in the concentration of orthodiphenols. Hydric stress induces changes in the activity of enzymes responsible for synthesizing phenolic compounds, such as *L-phenylalanine ammonia lyase*, which typically shows higher activity under water deficit [38]. Therefore, the observed variations in the total polyphenol and orthodiphenol content can be attributed to the different hydric stress levels experienced by the olive trees. These findings are consistent with the results of Servili et al. [39] and de Torres et al. [40], which showed that oils obtained from irrigated trees, i.e., Leccino, Frantoio, and Picual, had a lower concentration of these compounds compared to non-irrigated trees. Figure 4 shows the pigment content variations in oil samples at different ripeness stages.

**Figure 5:**
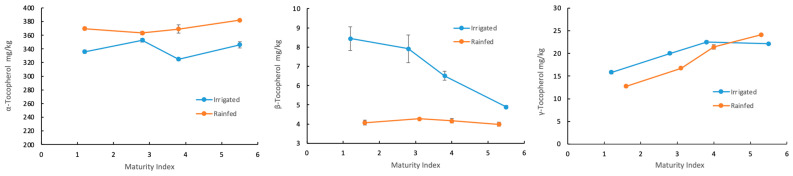
Tocopherol content in oils extracted from olives at different MI and grown under rainfed and irrigated conditions.

Differences in chlorophyll and carotene contents between rainfed and irrigated oils were evident across all ripening stages. The concentration of these compounds follows a similar pattern in relation to both the MI and the cultivation conditions. As in the previous case, the concentration of these compounds was higher for oils obtained from rainfed crops. The most significant differences between rainfed and irrigated oils were found in their tocopherol content (Figure 5).

The α-tocopherol content increased significantly in rainfed oils but was generally unaffected by the MI of the olives (Figure 5, left). In contrast, irrigation conditions positively affected the β- and γ-tocopherols contents. Trees subjected to hydric stress yielded oils with higher concentrations of these compounds. The concentration of β-tocopherol nearly doubled, whereas the increase in γ-tocopherol concentration was more moderate (Figure 5, right). Consequently, irrigation had no beneficial effect on the amount of antioxidant compounds, such as polyphenols, chlorophylls, carotenes, and α-tocopherol but may increase the content of β- and γ-tocopherols in the resulting olive oils.

## 4. Conclusions

This work provided relevant insights into understanding the relationship between agronomic factors, i.e., cultivation, olive maturation, and irrigation management, and the technological factors involved in the production process, on the antioxidant content of Picual virgin olive oil. Mathematical models were successfully applied to maximize the content of polyphenols, orthodiphenols, chlorophylls, carotenes, and α-, β-, and γ-tocopherols in olive oils. Increasing the malaxation temperature and reducing the malaxation time positively affected the content of total polyphenols and orthodiphenols. Moreover, increasing the malaxation temperature and mixing time positively impacted the levels of photosynthetic pigments. The α-tocopherol content was found to be more affected by the irrigation conditions than the MI. Talc dosage did not significantly affect the antioxidant content. Optimal conditions to produce antioxidant-enriched virgin olive oils include using olives from rainfed crops with an MI of 3–4 and a malaxation process of 60 min at 60 °C. Under these conditions, the total phenol content doubled, pigment content increased approximately fourfold, and the α-tocopherol content increased by 10–15%, while the β- tocopherol showed a slight increase. Monitoring the effects of climate change on olive oil quality can provide valuable insights into mitigating the consequences of drought and ensuring the resilience of this vital agricultural sector in the Mediterranean basin.

## Figures and Tables

**Table 1:** Experimental design for response surface analysis in terms of coded values and actual values.

Run	Temperature		Time		Talc Dosage	
	Coded Values	Actual Values (°C)	Coded Values	Actual Values (min)	Coded Values	Actual Values (%)
11	−1.68 *	6.4	0	40	0	1.25
6	−1	20	−1	20	−1	0.5
9	−1	20	−1	20	+1	2
2	−1	20	+1	60	−1	0.5
5	−1	20	+1	60	+1	2
17	0	40	−1.68 *	6.4	0	1.25
10	0	40	0	40	−1.68 *	0
3	0	40	0	40	0	1.25
7	0	40	0	40	0	1.25
8	0	40	0	40	0	1.25
15	0	40	0	40	0	1.25
13	0	40	0	40	+1.68 *	2.5
1	0	40	+1.68 *	73.6	0	1.25
16	+1	60	−1	20	−1	0.5
12	+1	60	−1	20	+1	2
14	+1	60	+1	60	−1	0.5
18	+1	60	+1	60	+1	2
4	+1.68 *	70	0	40	0	1.25

* Axial points.

**Table 2:** Compositional features of processed olive fruits.

Harvest Dates	Irrigation Management	Maturity Index	Moisture (g/kg)	Oil (g/kg)	Solids (g/kg)
October	Rainfed	1.6	552 ± 4	175 ± 2	273 ± 3
October	Rainfed	3.1	493 ± 3	215 ± 3	292 ± 6
November	Rainfed	4.0	461 ± 11	250 ± 7	289 ± 7
December	Rainfed	5.3	426 ± 8	256 ± 10	319 ± 11
October	Irrigated	1.2	563 ± 15	175 ± 3	262 ±14
October	Irrigated	2.8	538 ± 8	207 ± 4	256 ± 7
November	Irrigated	3.8	507 ± 10	233 ± 5	260 ± 7
December	Irrigated	5.5	438 ± 11	272 ± 4	290 ± 8

**Table 3:** Total phenols, photosynthetic pigments, and tocopherols composition in oils obtained from irrigated olive trees and fruits with a maturity index of 3.8.

Run	Phenolic Compounds (mg/kg)		Pigments (mg/kg)		Tocopherols (mg/kg)		
	Phenols	Orthodiphenols	Chlorophylls	Carotenes	α-Tocopherol	β-Tocopherol	γ-Tocopherol
1	524	62.9	39.3	13.1	325.9	5.60	22.9
2	417	60.2	33.9	11.1	323.1	3.15	21.6
3	571	91.6	41.3	14.3	330.7	3.79	20.1
4	800	197.5	66.1	21.3	318.0	6.45	18.8
5	420	61.8	35.5	11.6	328.2	2.84	19.8
6	603	94.1	35.7	11.2	323.8	2.05	18.3
7	586	99.1	42.2	14.8	321.4	3.77	18.5
8	565	91.4	41.5	13.9	312.6	3.34	19.1
9	572	86.3	34.3	11.4	317.0	3.71	19.5
10	575	86.5	36.2	12.3	315.5	4.14	19.3
11	394	84.6	32.1	10.1	319.1	3.32	18.6
12	686	149.2	57.4	19.4	333.6	5.40	19.2
13	562	79.0	40.4	13.6	315.2	3.92	18.9
14	734	139.6	59.4	21.3	314.3	6.12	18.9
15	571	94.1	39.7	13.5	310.4	3.87	18.8
16	658	118.5	48.6	16.6	311.5	5.20	18.8
17	588	86.5	35.5	11.7	317.1	3.57	19.0
18	749	145.2	70.2	23.0	324.4	6.51	18.5

**Table 4:** Total phenols, photosynthetic pigments, and tocopherols composition from oils obtained from rainfed olive trees and fruits with a maturity index of 4.

Run	Phenolic Compounds (mg/kg)		Pigments (mg/kg)		Tocopherols (mg/kg)		
	Phenols	Orthodiphenols	Chlorophylls	Carotenes	α-Tocopherol	β-Tocopherol	γ-Tocopherol
1	551	83.1	55.2	20.3	369.1	5.15	19.8
2	459	56.6	34.6	11.3	323.7	3.53	19.9
3	551	93.3	51.1	18.8	353.6	3.92	18.1
4	696	183.9	68.3	23.1	344.0	3.87	21.4
5	471	68.7	34.0	11.0	322.1	3.90	20.3
6	524	78.9	34.3	11.1	287.8	5.21	19.6
7	548	88.2	44.8	16.9	345.8	3.86	18.9
8	541	88.8	46.5	17.7	335.8	3.86	18.9
9	575	85.5	33.0	10.3	319.1	3.73	19.8
10	506	94.8	48.7	18.5	360.1	4.27	18.5
11	393	69.5	30.7	10.3	326.6	4.10	20.2
12	734	128.0	54.5	19.1	344.9	4.08	20.4
13	509	91.3	44.9	16.2	340.2	3.91	19.9
14	651	146.4	68.1	23.1	340.5	3.83	20.5
15	524	81.2	45.3	17.2	341.9	3.46	19.9
16	684	133.4	54.9	19.7	338.7	3.87	20.5
17	676	105.3	43.4	15.7	362.8	3.46	18.4
18	624	154.5	74.1	25.4	354.1	3.82	22.2

**Table 5:** Maximum values for each response of total phenols and orthodiphenols.

		Phenols				Orthodiphenols			
Irrigation Management	MI	Max. Value (mg/kg)	Temperature (°C)	Time (min)	Talc (%)	Max. Value (mg/kg)	Temperature (°C)	Time (min)	Talc (%)
Irrigated	1.2	725.70 ± 1.67	60	30.41	0.50	91.22 ± 1.67	60	32.59	1.22
	2.8	760.90 ± 18.2	60	60	0.96	129.28 ± 3.31	60	60	0.50
	3.8	749.87 ± 9.91	60	60	2	154.83 ± 3.24	60	38.23	2
	5.5	533.04 ± 8.77	46.99	29.56	1.39	109.99 ± 4.43	60	37.78	0.51
Rainfed	1.6	760.02 ± 18.9	60	20	0.54	124.06 ± 3.61	60	60	0.5
	3.1	771.72 ± 13.7	60	59.6	2	175.02 ± 7.95	60	60	2
	4.0	723.47 ± 15.6	60	20.04	2	150.55 ± 5.54	60	60	1.36
	5.3	730.12 ± 16.5	60	60	2	136.14 ± 3.62	60	48.76	1.11

**Table 6:** Maximum values for each response of chlorophylls and carotenes.

		Chlorophylls				Carotenes			
Irrigation Management	MI	Max. Value (mg/kg)	Temperature (°C)	Time (min)	Talc (%)	Max. Value (mg/kg)	Temperature (°C)	Time (min)	Talc (%)
Irrigated	1.2	112.02 ± 3.87	60	60	1.50	28.53 ± 0.73	60	60	1.23
	2.8	83.32 ± 1.96	60	60	0.82	22.75 ± 0.52	60	60	2
	3.8	66.77 ± 2.80	60	60	2	22.89 ± 0.23	60	60	2
	5.5	38.40 ± 0.77	59.8	20.11	2	13.70 ± 0.29	60	60	2
Rainfed	1.6	117.16 ± 2.96	60	60	1.75	36.63 ± 0.74	60	60	2
	3.1	85.64 ± 1.95	60	60	0.51	26.42 ± 1.56	60	60	0.59
	4.0	73.42 ± 2.20	60	60	2	24.30 ± 0.84	60	60	1.69
	5.3	47.26 ± 1.46	60	60	0.5	17.74 ± 0.46	60	60	0.50

**Table 7:** Maximal values for each response of α-, β-, and γ-tocopherol.

		α-Tocopherol				β-Tocopherol				γ-Tocopherol			
Irrigation Management	MI	Max. Value (mg/kg)	T (°C)	Time (min)	Talc (%)	Max. Value (mg/kg)	T (°C)	Time (min)	Talco (%)	Max. Value (mg/kg)	T (°C)	Time (min)	Talco (%)
Irrigated	1.2	335.9 ± 2.48	46	60	0.5	8.44 ± 0.62	60	20	2	15.81 ± 0.31	20	60	0.67
	2.8	352.72 ± 2.83	59.4	60	2	7.92 ± 0.72	20	20	2	20.10 ± 0.23	60	35.3	1.44
	3.8	324.86 ± 2.20	20	60	0.5	6.51 ± 0.24	59.8	60	2	22.51 ± 0.31	60	60	0.5
	5.5	346.06 ± 4.70	59.8	60	0.5	4.89 ± 0.07	60	53.7	2	22.14 ± 0.26	60	60	2
Rainfed	1.6	369.89 ± 2.61	38.2	46.7	1.7	4.06 ± 0.13	20	43.1	0.5	12.73 ± 0.21	20.9	34.03	2
	3.1	363.15 ± 2.65	60	60	2	4.27 ± 0.05	60	60	0.5	16.74 ± 0.14	60	46.3	0.5
	4.0	369.10 ± 6.01	55.9	57.9	1.9	4.17 ± 0.11	60	60	0.5	21.42 ± 0.49	60	20	2
	5.3	382.01 ± 0.1	52.4	60	1.4	3.99 ± 0.10	44.5	60	0.5	24.10 ± 0.20	59.4	20	2

## Data Availability

The original contributions presented in the study are included in the article/Supplementary Materials, further inquiries can be directed to the corresponding author.

## Supplementary Materials

The following supporting information can be downloaded at: https://www.mdpi.com/article/10.3390/foods13132093/s1, Figure S1: Response surfaces of (a) phenols (rainfed—M.I 3.1), (b) chlorophylls (irrigation—M.I 3.8), (c) carotenes (irrigation—M.I 3.8), (d) α-tocopherol (rainfed—MI 5.3). Table S1: Models obtained with actual factors and statistical parameters for the response surface of virgin olive oils derived from irrigated oils. Table S2: Models obtained with actual factors and statistical parameters for the response surface of virgin olive oils derived from rainfed olives.
