# Supplementary material for: Maximizing Antioxidant Potential in Picual Virgin Olive Oil: Tailoring Agronomic and Technological Factors with Response Surface Methodology
Source: Foods. 2024 Jul 1;13(13):2093. doi: 10.3390/foods13132093 (PMC11241712; doi:10.3390/foods13132093)
Supplement: Supplementary file 1 [file foods-13-02093-s001.zip › foods-3046771-supplementary.pdf]

Supplementary Materials

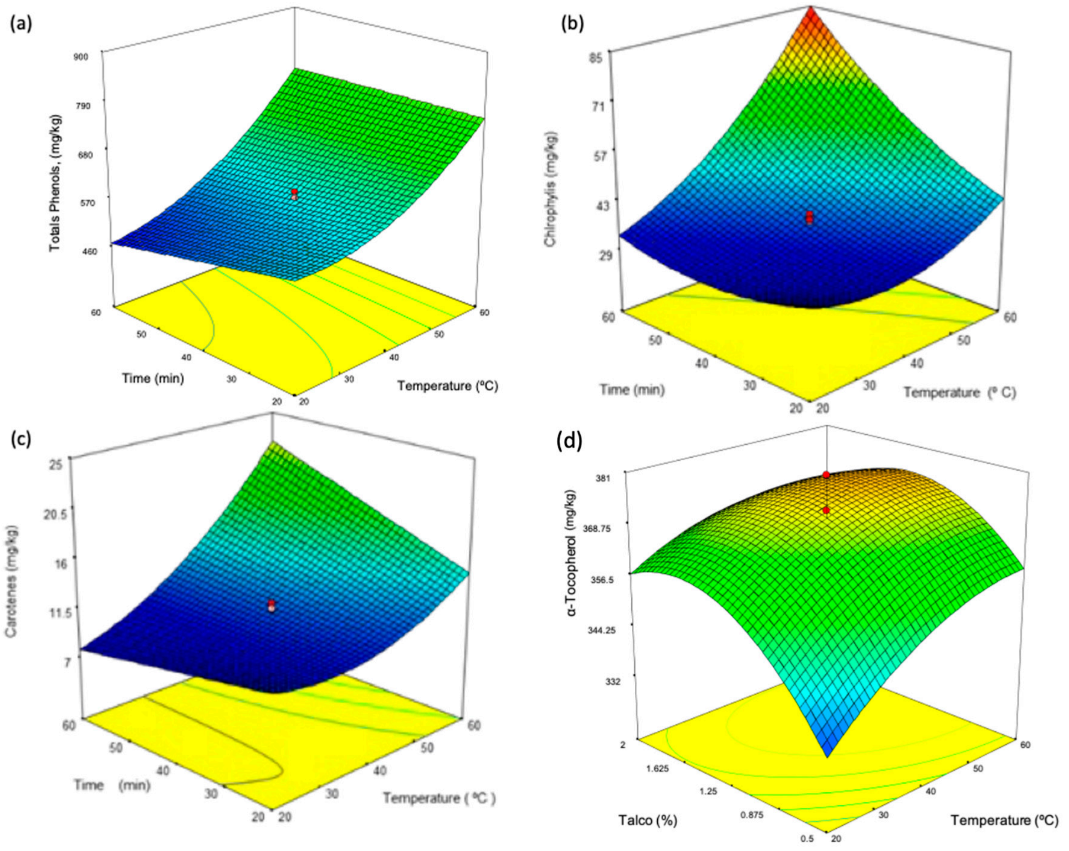

Figure S1. Response surfaces of (a) phenols (Rainfed – M.I 3.1), (b) chlorophylls (Irrigation – M.I 3.8), (c) carotenes (Irrigation – M.I 3.8), (d) α-tocopherol (Rainfed – MI 5.3).

Table S1. Models obtained with actual factors and statistical parameters for the response surface of virgin olive oils derived from irrigated oils.

Response (mg/kg)	Model	p-value	R^2	Std. Dev.
**MI: 1.2**				
Phenols	$601.0 + 103.8\ T - 25.9\ t - 2.4\ D - 10.4\ T\ D + 8.4\ t\ D - 35.6\ t^2$	< 0.0001	0.9925	10.4
Orthodiphenols	$58.8 + 17.9\ T - 6.6\ t - 0.2\ D + 13.3\ T^2 - 8.9\ t^2 - 1.8\ D^2$	< 0.0001	0.9964	1.67
Chlorophylls	$58.9 + 25.4\ T + 11.9\ t + 1.5\ D + 11.2\ T\ t + 4.36\ T^2$	< 0.0001	0.9867	3.87
Carotenes	$18.2 + 7.2\ T + 1.7\ t + 0.5\ D + 1.5\ T\ t - 0.53\ t\ D + 1.1\ T^2 - 1.1\ t^2 - 0.9\ D^2$	< 0.0001	0.9929	0.73
α – Tocopherol	$329.5 + 3.3\ T + 4.1\ t + 2.6\ D - 4.5\ t\ D - 5.6\ T^2$	< 0.0001	0.9419	2.48
β – Tocopherol	$4.2 + 1.3\ T + 0.2\ t + 1.2\ D - 0.9\ T\ t - 1.2\ T\ D + 0.8\ T^2 - 0.8\ D^2$	< 0.0001	0.9462	0.62
γ - Tocopherol	$11.6 + 0.3\ T + 2.3\ t - 0.4\ T\ t + 0.6\ T^2 + 1.2\ t^2$	< 0.001	0.9818	0.31
**MI: 2.8**				

Phenols	$533.3 + 82.4\ T - 514.6\ t + 67.3\ D + 75.9\ T\ t + 29.6\ T\ D - 111.6\ t\ D + 24.8\ T^2 - 19.5\ D^2$	< 0.0001	0.9913	18.2
Orthodiphenols	$74.3 + 26.5\ T - 15.6\ t - 6.7\ D + 36.2\ T\ t + 27.1\ T\ D - 27.8\ t\ D + 5.7\ T^2 - 2.0\ t^2 - 3.2\ D^2$	< 0.0001	0.9962	3.21
Chlorophylls	$37.4 + 15.0\ T + 9.62\ t + 0.09\ D + 10.8\ T\ t + 1.84\ t\ D + 7.54\ T^2 + 4.45\ t^2$	< 0.0001	0.9911	1.96
Carotenes	$11.7 + 4.59\ T + 1.03\ t + 0.40\ D + 2.66\ T\ t - 1.08\ T\ D + 1.21\ t\ D + 2.23\ T^2$	< 0.0001	0.9918	0.52
α – Tocopherol	$336.4 - 1.01\ T + 8.62\ t + 6.83\ D + 5.85\ T\ t + 5.04\ T\ D - 5.07\ T^2 - 3.88\ t^2$	< 0.0001	0.9544	2.83
β – Tocopherol	$4.06 - 0.27\ T - 2.09\ t - 0.96\ D - 2.87\ T\ D - 1.40\ t\ D + 1.19\ D^2$	< 0.0002	0.9541	0.72
γ - Tocopherol	$19.7 + 0.37\ T - 0.18\ t + 0.16\ D - 0.37\ t^2 - 0.32\ D^2$	< 0.0028	0.8917	0.23
**MI: 3.8**				
Phenols	$572.1 + 100.9\ T - 22.5\ t - 0.49\ D + 59.6\ T\ t + 8.87\ T\ D + 36.3\ T^2 - 4.96\ t^2$	< 0.0001	0.9948	9.91
Orthodiphenols	$93.0 + 39.1\ T - 8.66\ t - 1.45\ D + 14.1\ T\ t + 9.97\ T\ D - 6.62\ t\ D + 19.9\ T^2 - 6.64\ t^2 - 3.85\ D^2$	< 0.0001	0.9963	3.24
Chorophylls	$40.5 + 11.9\ T + 2.15\ t + 1.98\ D + 3.03\ T\ t + 2.43\ T\ D + 4.81\ T^2$	< 0.0001	0.9624	2.80
Carotenes	$14.1 + 4.10\ T + 1.05\ t + 0.54\ D + 1.02\ T\ t + 0.47\ T\ D + 0.81\ T^2 + 1.21\ t^2 - 0.41\ D^2$	< 0.0001	0.9882	0.66
α - Tocopherol	$311.6 - 0.74\ T + 1.53\ t + 0.57\ D + 4.01\ T\ D + 2.66\ T^2 + 3.52\ t^2 + 1.35\ D^2$	< 0.0081	0.9102	2.20
β – Tocopherol	$3.73 + 1.12\ T + 0.57\ t + 0.46\ D - 0.37\ T\ D + 4.44\ T^2 + 0.25\ t^2 + 0.32\ D^2$	< 0.0001	0.9827	0.24
γ – Tocopherol	$18.8 + 0.16\ T + 1.10\ t - 0.22\ D - 0.29\ T\ t - 0.60\ T\ D - 1.16\ t\ D + 0.74\ t^2$	< 0.0001	0.9679	0.31
**MI: 5.5**				
Phenols	$508.6 + 36.1\ T - 30.6\ t + 1.73\ D + 5.78\ T\ D - 53.3\ T^2 - 29.3\ t^2 - 10.1\ D^2$	< 0.0001	0.9895	8.77
Ortodiphenols	$94 + 23.1\ T - 5.64\ t + 4.04\ T\ t - 7.21\ T^2 - 7.18\ t^2$	< 0.0001	0.9765	4.43
Chorophylls	$31.7 + 3.10\ T - 0.36\ t - 0.70\ D + 1.38\ T\ t + 2.28\ T\ D - 1.0\ t\ D + 1.42\ t^2 + 0.64\ D^2$	< 0.0002	0.9774	0.77
Carotenes	$10.9 + 1.07\ T + 0.74\ t + 0.02\ D + 0.22\ T\ t - 0.19\ T^2 + 0.71\ t^2 + 0.24\ D^2$	< 0.0001	0.9714	0.29
α - Tocopherol	$317.7 - 2.54\ T + 4.30\ t - 3.83\ D + 9.39\ Tt - 11.1\ T\ D + 6.46\ t\ D + 4.11\ T^2 + 4.89\ D^2$	< 0.0124	0.9084	9.60
β - Tocopherol	$3.68 + 0.45\ T - 0.40\ t + 0.48\ D + 0.58\ T\ t - 0.21\ T\ D + 0.34\ t\ D + 0.14\ T^2 - 0.34\ t^2$	< 0.0001	0.9652	0.07
γ - Tocopherol	$20.8 + 0.79\ T + 0.16\ t - 0.06\ D - 0.54\ T\ t + 0.85\ T\ D + 0.66\ t\ D - 0.27\ T^2 - 0.24\ D^2$	< 0.0070	0.9253	0.26

T: malaxation temperature (°C), t: malaxation time (min), D: talc dosage (%), R^2: coefficient of determination, Std. Dev.: standard deviation

Table S2. Models obtained with actual factors and statistical parameters for the response surface of virgin olive oils derived from rainfed olives.

Response (mg/kg)	Model	p-value	R^2	Std. Dev.
**MI: 1.6**				
Totals phenols	$393.1 + 79.4\ T - 37.9\ t + 153.5\ T^2 + 94.0\ t^2$	< 0.0001	0.9895	18.9
Orthodiphenols	$67.7 + 33.4\ T - 4.23\ t - 0.37\ D + 12.5\ T\ t - 2.61\ t\ D + 19.9\ T^2 - 2.11\ t^2$	< 0.0001	0.9933	3.61
Chlorophylls	$47.9 + 24.5\ T + 13.5\ t + 1.65\ D + 14.2\ T\ t + 1.79\ T\ D + 11.8\ T^2 + 3.23\ t^2$	< 0.0001	0.9937	2.96
Carotenes	$16.1 + 7.54\ T + 4.07\ t + 0.41\ D + 4.19\ T\ t + 0.51\ T\ D + 3.39\ T^2 + 0.60\ t^2$	< 0.0001	0.9957	0.74

α – Tocopherol	$367.9 – 2.85 T + 5.40 t + 2.83 D + 2.68 T D – 6.55 T^2 – 8.11 t^2 – 2.06 D^2$	< 0.0001	0.9657	2.61
β – Tocopherol	$3.90 – 0.12 T + 0.12 t – 0.17 D – 0.18 T t + 0.19 TD + 0.25 t D – 0.16 T^2 -0.14 t^2 – 0.15 D^2$	< 0.0062	0.9350	0.13
γ - Tocopherol	$12.5 – 0.28 T + 0.19 t – 0.02 D + 0.16 T t – 0.18 T D – 0.19 t D – 0.27 T^2 – 0.25 t^2$	< 0.0127	0.9076	0.21
**MI: 3.1**				
Totals phenols	$578.6 + 119.9 T – 23.6 t + 1.77 D + 22.6 T t + 12.9 T D + 13.1 t D + 54.6 T^2 – 8.05 D^2$	< 0.0001	0.9925	13.71
Orthodiphenols	$74.7 + 25.7 T – 11.9 t – 6.93 D + 19.0 T t + 17.3 T D + 16.3 t D + 34.5 T^2 + 6.40 t^2$	< 0.0001	0.9830	7.95
Chlorophylls	$42.6 + 14.8 T + 7.55 t + 8.10 T t + 7.82 T^2 + 4.83 t^2$	< 0.0001	0.9915	1.94
Carotenes	$15.0 + 4.52 T + 1.53 t + 2.32 T t + 3.03 T^2$	< 0.0001	0.9338	1.56
α – Tocopherol	$353.1 + 0.35 T + 7.47 t + 3.81 D + 6.36 T t – 2.93 T^2 – 3.12 t^2 – 1.85 D^2$	< 0.0001	0.9490	2.65
β – Tocopherol	$3.69 + 0.11 T + 0.08 t – 9.61 10^{-3} D + 0.05 T t – 0.12 T D + 0.10 T^2 + 0.11 t^2$	< 0.0007	0.9427	0.05
γ - Tocopherol	$17.9 + 0.26 T + 0.16 t – 0.04 D – 0.41 T D + 0.09 T^2 – 0.25 t^2$	< 0.0004	0.9464	0.14
**MI: 4.0**				
Totals phenols	$535.3 + 97.4 T – 38.3 t + 6.71 D – 14.5 t D + 25.2 T^2 + 29.0 t^2 – 8.65 D^2$	< 0.0001	0.9805	15.6
Orthodophenols	$89.6 + 35.9 T – 0.97 t + 9.83 T t + 16.2 T^2$	< 0.0001	0.9811	5054
Chorophylls	$47.7 + 12.9 T + 3.36 t – 0.79 D + 4.95 T t + 1.95 T D + 1.90 t D + 1.46 T^2$	< 0.0001	0.9832	2.20
Carotenes	$17.6 + 5.46 T + 1.34 t + 1.10 T t – 1.25 T^2$	< 0.0001	0.9740	0.84
α - Tocopherol	$346.2 + 4.33 T + 1.60 t – 5.63 D + 23.4 T t + 18.9 T D + 23.7 t D – 4.19 T^2 + 6.97 t^2$	< 0.0054	0.9320	6.01
β – Tocopherol	No significative			
γ – Tocopherol	$18.6 + 0.52 T + 0.36 t + 0.33 D + 1.01 T^2 + 0.26 t^2 + 0.33 D^2$	< 0.0008	0.8645	0.49
**MI: 5.3**				
Totals phenols	$446.6 + 148.8 T – 12.0 t + 5.44 D + 31.34 T t + 19.2 T D + 22.4 t D + 55.4 T^2 + 13.0 t^2$	< 0.0001	0.9918	16.5
Ortodiphenols	$91.5 + 30.6 T – 4.76 t – 2.29 D + 6.75 T t + 13.4 T^2 – 2.26 t^2 – 6.17 D^2$	< 0.0001	0.9925	3.62
Chorophylls	$34.7 + 6.97 T + 0.81 t – 0.80 D + 1.73 Tt + 2.22 T^2$	< 0.0001	0.9882	1.46
Carotenes	$12.9 + 2.82 T + 0.32 t – 0.28 D + 0.72 T t + 0.73 T^2$	< 0.0001	0.9805	0.46
α - Tocopherol	$372.0 + 8.34 T + 6.88 t + 7.66 D – 4.42 T D – 6.01 T^2 – 12.6 D^2$	< 0.0001	0.9478	4.68
β - Tocopherol	$3.74 – 0.17 T + 0.27 t – 0.02 D + 0.26 T t – 0.10 t D – 0.19 T^2- 0.14 t^2$	< 0.0015	0.9519	0.10
γ - Tocopherol	$22.3 + 0.79 T – 0.43 t + 0.06 D – 0.53 t D – 0.34 T^2 + 0.38 t^2$	< 0.0001	0.9559	0.20

T: malaxation temperature (°C), t: malaxation time (min), D: dosage of talc (%), R^2: coefficient of determination, Std. Dev.: standard deviation
